# The impact of changing toward higher welfare broiler production systems on greenhouse gas emissions: a Dutch case study using life cycle assessment

**DOI:** 10.1016/j.psj.2022.102151

**Published:** 2022-08-27

**Authors:** P.F. Mostert, A.P. Bos, J. van Harn, I.C. de Jong

**Affiliations:** Wageningen Livestock Research, Wageningen University and Research, PO Box 338, 6700 AH Wageningen, the Netherlands

**Keywords:** environmental impact, soybean, slower grower broiler breed, land use change, animal welfare

## Abstract

In the Netherlands, the Dutch Retail Broiler (**DRB**) and Better Life one Star (**BLS**) production systems have been introduced with the aim to improve broiler welfare. Simultaneously, retailers set targets for reduction of greenhouse gas (**GHG**) emissions in the whole broiler production chain. The GHG emissions of DRB and BLS may differ from conventional systems because of differences in slaughter age, feed intake, and diet composition. The aim of this study was to estimate GHG emissions of the conventional, DRB, and BLS production systems. A deterministic, spreadsheet based model was developed that included the breeder, hatchery, and broiler farm stages. First, the model calculates feed intake of different diets and energy use, based on performance objectives and literature. Selection of feed ingredients for the different types of diets was based on least cost formulation with nutritional constraints for each diet. Second, GHG emissions were estimated from cradle to broiler farm gate for processes along the broiler production chain by using life cycle assessment, and expressed as kg CO_2_-equivalents per kg live weight (kg CO_2_-eq/kg LW). Results showed that BLS (3.55 kg CO_2_-eq/kg LW) had lower GHG emissions compared to conventional (3.65 kg CO_2_-eq/kg LW) and DRB (3.98 kg CO_2_-eq/kg LW) at the broiler farm gate. Emissions from land use change (**LUC**) from feed production, mainly from soybean products, had highest impact on total GHG emissions (>50%) for the systems and these soybean products had the lowest inclusion in the diets of the BLS production system. Sensitivity analyses showed that variation in slaughter weight and feed intake could result in overlap of GHG emissions between systems. When soybean products were sourced from a country with low LUC emissions, conventional (1.37 kg CO_2_-eq/ kg LW) had the lowest GHG emissions and BLS (1.79 kg CO_2_-eq/kg LW) the highest. This study showed that origin of and including or excluding LUC emissions from soybean production results in different conclusions for achieving the GHG emissions reduction targets set by retailers.

## INTRODUCTION

The conventional broiler production system is currently the most adopted in the European Union (BCC, 2020). However, the conventional broiler production system, using fast growing broilers at relatively high stocking densities, is being criticized because of the negative impact on broiler welfare (Bessei, 2006; Vissers et al., 2019; BCC, 2020). Therefore, there is an increasing interest in western European countries towards the implementation of so-called ‘higher-welfare’ broiler production systems (ECC, 2018). Examples of ‘higher-welfare’ production systems are the Dutch Retail Broiler (**DRB**) and Better Life one Star (**BLS**) broiler production systems that have been introduced in the Netherlands with the aim to improve broiler welfare as compared to the conventional broiler production system (Saatkamp et al., 2019). The DRB and BLS production systems use broiler breeds with a lower average daily growth rate (<50 g/day) compared to the conventional breeds (>60 g/day), and provide more space per chicken than conventional production system (Saatkamp et al., 2019). BLS has more strict welfare requirements as compared to DRB, and also has a covered outdoor run (Vissers et al., 2019). De Jong et al. (2022) showed that DRB and BLS production systems had a higher average total welfare score on flock level compared to conventional. From 2023 onward, Dutch retailers will source their fresh broiler meat only from BLS production systems (Dierenbescherming, 2021). Simultaneously, some Dutch retailers want to reduce greenhouse gas (**GHG**) emissions in the broiler production chain by 15% in 2030 compared to 2018, while keeping the same income for farmers (AH, 2021). Van Horne (2020) showed that Dutch farmers with the DRB and BLS production systems have higher costs, but also higher revenues and therefore they have a similar income compared to farmers with a conventional broiler production system. However, so far little attention has been paid to the impact of the DRB and BLS production systems on GHG emissions. Reducing GHG emissions is not only relevant for the Dutch production chain, but also for the worldwide poultry sector in the light of international and national climate agreements, that is, climate neutral in Europe and the Netherlands in 2050, and even more relevant if the expected increase in poultry meat consumption in the next decades (FAO, 2018) is taken into account.

Life cycle assessment (**LCA**) is a method to estimate the impact of a product on the environment. An LCA includes all processes and related environmental impacts in the entire life cycle of a product (Baumann and Tillmann, 2004) and is a common method to estimate GHG emissions of broiler meat (De Vries and de Boer, 2010). A global assessment of GHG emissions of broiler meat production showed that most GHG emissions are from feed production (57%) and land use change (**LUC**) from feed production (21%) (Gerber et al., 2013). Several LCA studies were performed to estimate GHG emissions of conventional broiler production in different countries (González-García et al., 2014; Putman et al., 2017; Benavides et al., 2020). These studies, however, did not compare different broiler production systems. Leinonen et al. (2012) compared GHG emissions of conventional, free range, and organic broiler chickens in the United Kingdom by using LCA. They showed that conventional broilers had the lowest impact on GHG per expected edible carcass weight because of the lowest feed conversion rate **(FCR)**. Tallentire et al. (2018) showed that GHG emissions of producing the total feed required for slower grower broilers (growth rate 38.6 g/day) increases by 27% compared to current broilers due to a higher feed intake. However, these latter studies did not give insight in how indoor ‘higher-welfare’ systems, such as DRB and BLS, compare to the conventional production system. The GHG emissions of BLS and DRB may differ from Dutch conventional systems because of differences in slaughter age, FCR, mortality, and diet composition. Insight in the GHG emissions of these ‘higher-welfare’ systems as compared to the conventional system is relevant in light of the expected increase in these indoor higher welfare systems due to developments such as Better Chicken Commitment (BCC, 2020).

Therefore, the objective of this study was to estimate GHG emissions of the conventional, DRB, and BLS broiler production systems from cradle to broiler farm gate for the Netherlands. Our work is a case study that estimates GHG emissions by using an attributional LCA. Estimating the impact of the DRB and BLS production systems on GHG emissions can show whether there are trade-offs or synergies between Dutch broiler production systems with higher broiler welfare and GHG emissions. This information is important for future development of sustainable broiler production systems.

## MATERIALS AND METHODS

**Figure 1:**
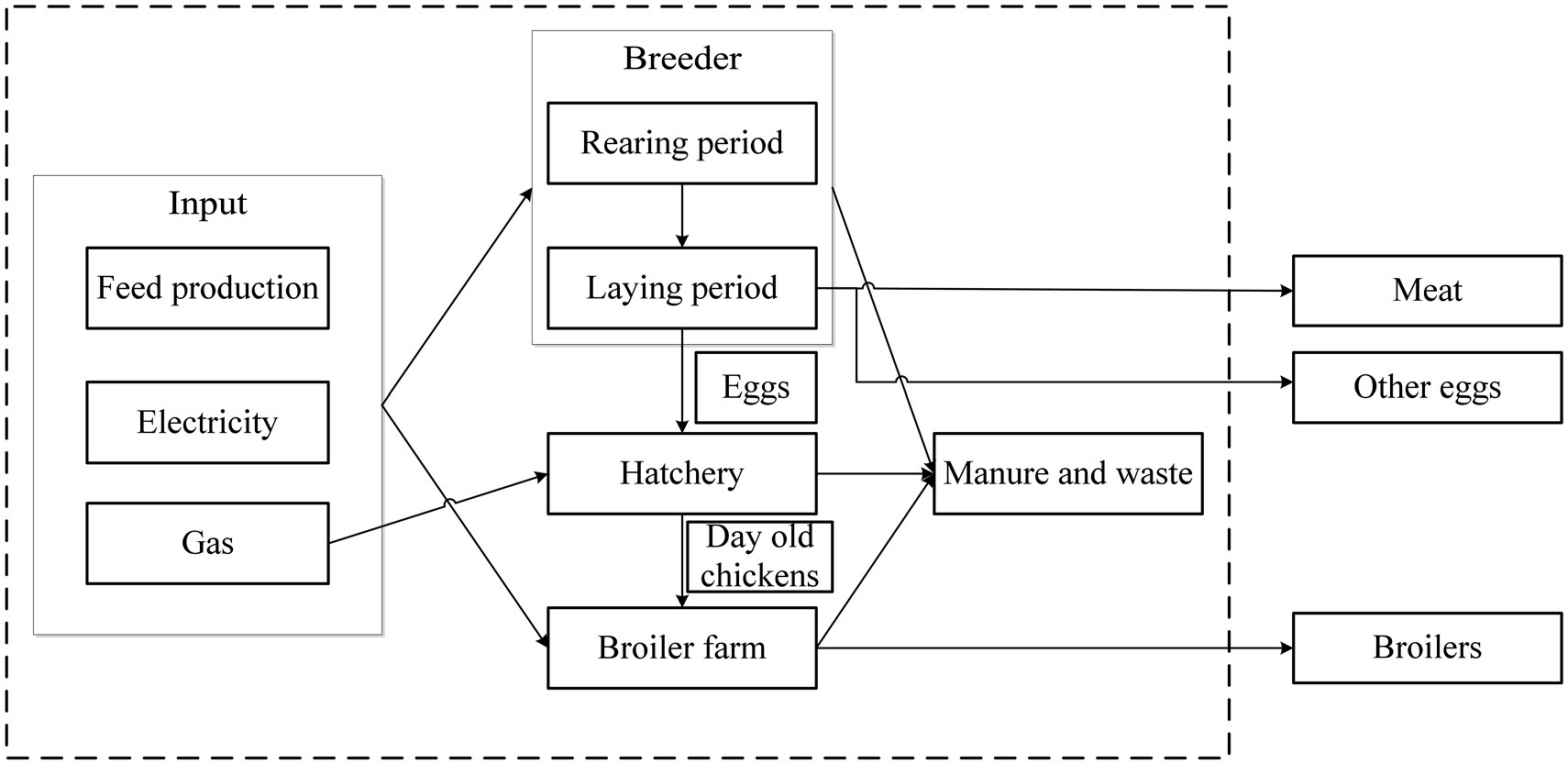
Overview of main input, output and stages of the broiler production chain included to estimate greenhouse gas emissions of different broiler production systems.

A deterministic, spreadsheet based model was developed to estimate GHG emissions of three Dutch broiler production systems: conventional, DRB, and BLS. The production systems analyzed in our study include the breeder, hatchery, and broiler farm stage and input for these stages (Figure 1). The breeder stage consisted of 2 periods: rearing period (0–20 wk) and laying period (>20 wk of age). The model consists of 2 parts. The first part calculates the production parameters of the breeder, hatchery and broiler farm stage based on performance objectives from industry and literature. The second part estimates GHG emissions for each stage by using an LCA. Production parameters and GHG emissions are expressed per hen (rearing period), per egg delivered to hatchery (laying period), per egg hatched at hatchery (hatchery), and per kg live weight (broiler farm). Compared to conventional conditions for production, DRB and BLS have different conditions in the broiler farm stage, for example, a longer production period, a lower stocking density (Table 1), and provision of environmental enrichment in the house. The BLS system also prescribes a covered outdoor run (wintergarten). There are no differences in conditions for production in the breeder stage for the different broiler production systems. The different broiler production systems were analyzed with the most common broiler breed used in the particular system in the Netherlands in 2018: for conventional the Ross 308, for DRB the Ranger Classic, for BLS the Hubbard JA257 (Personal communication Aviagen, Plukon Food Group).

**Table 1:** Broiler farm conditions and input to estimate greenhouse gas emissions of conventional, Dutch Retail Broiler (DRB), and Better Life one Star (BLS) production systems.

Input	Conventional	DRB	BLS
Production period (d)^a^	38	49	56
Empty period (d)^b^	7	7	7
Density, maximum (kg/m^2^)^b^	42	38	25
Electricity per chicken housed (kWh)^c^	0.17	0.22	0.27
Gas per chicken housed (m^3^)^c^	0.07	0.10	0.13
Slaughter weight (g)^a^	2,527	2,449	2,389
Mortality and selection (%)^b^	3.5	3.0	2.5
Feed intake a			
Starter (g/chicken/cycle)	304	244	373
Grower 1 (g/chicken/cycle)	771	585	783
Grower 2 (g/chicken/cycle)	1,340	981	1,029
Finisher (g/chicken/cycle)	1,457	2,650	2,799
Total feed intake per chicken (kg)	3.87	4.46	4.98
FCR^a^^,^^d^ (kg feed/kg slaughter weight)	1.53	1.82	2.09

a Hubbard 2017; Aviagen, 2018; Aviagen, 2019.

b KWIN-V, 2018.

c Van Horne, 2020.

d Feed conversion rate.

### Production Parameters of the Breeder, Hatchery, and Broiler Farm Stage

Production parameters calculated were feed intake of different diets and energy use. These production parameters were calculated for the breeder, hatchery, and broiler farm stage.

#### Type of Diets, Feed Intake, and Selection of Feed Ingredients

Type of diets and feed intake of chickens of these diets at the different stages were based on recommendations and performance objectives of the breeding companies (Appendix A, Table A.1–A.7, Table 1). Selection of feed ingredients for each diet at the different stages for each production system were based on least cost formulation with nutritional constraints, as this is common practice, using a third-party linear programming tool (Adifo, 2020) (Appendix A, Tables A.1–A.7). Nutritional constraints were based on recommendations of the breeding companies for diet composition and performance objectives and prices of feed ingredients were average prices of the years 2017 and 2018 (LEI, 2019). At the breeder stage, only one rearing diet for the rearing period and one breeder diet for the laying period were formulated for every production system. In practice, 3 or 4 rearing or breeder diets are used in these periods (Aviagen, 2016; Hubbard, 2019) (Appendix A, Tables A.1–A.2). In this study the diet composition of the grower diet was used for the full rearing and the diet composition of breeder one diet for the full breeder period because these diets had the highest feed intake of the total feed intake during these periods (Appendix A, Tables A.1 and A.2). Males and females received the same diet compositions. Number of males in the laying period was estimated using the mating ratio (% male/female housed) (Appendix, Table A.2). Nutritional recommendations for breeds in the breeder stage of conventional and DRB production systems are the same (Appendix A, Table A.3), and therefore, these breeds have the same diet composition. At the broiler farm stage, 4 diets (starter, grower 1, grower 2, and finisher) for every broiler breed in each production system were formulated (Appendix A, Tables A.5–A.7).

#### Energy Use

Energy use (electricity and natural gas) in the breeder and hatchery stage was calculated based on energy prices (0.13€/kWh and 0.55€/m^3^ natural gas) and total costs of energy in 2018 (Appendix A, Tables A.1, A.2, A.4). In the breeder stage, data about energy costs was only available for the conventional production system and was assumed to be equal for the other production systems. In the laying period, the breed in BLS production system had a longer production period (65 wk instead of 60 wk) in comparison to the breeds in conventional and DRB production systems. Therefore, the energy use per male and female housed in BLS system was increased linearly with the length of weeks. Energy use in the broiler farm stage (Table 1) was based on Van Horne (2020). In the hatchery stage, it was assumed that energy costs per egg were similar between the broiler production systems and energy use was from natural gas.

#### Correction of Feed Intake and Energy Use

Input of feed intake and energy use were per living bird at the breeder and broiler farm stage and did not account for selection (injured or sick birds) and mortality (dead birds) (Appendix A, Tables A.1 and A.2; Table 1). Therefore, feed intake and energy use were corrected for selection and mortality. In addition, it was assumed that selected and dead birds had consumed feed before they were removed. However, no data from literature or industry were available about feed intake of the different diets of the selected and dead birds. In the breeder stage, the parents had only one type of diet and it was assumed that selected and dead birds were removed in the middle of the breeder stage. Therefore, in the breeder stage feed intake of the selected and dead birds was half of the total feed intake. In the broiler farm stage, every broiler in each production systems had 4 diets. Feed intake of the broilers and the composition of the 4 diets in the broiler farm stage were different between the production systems. Therefore, in the broiler farm stage, it was assumed that selected and dead broilers were removed at 4 different days evenly distributed over the production period for every production system (e.g., for conventional at d 5, 15, 25, 35). The number of selected and dead broilers for each of these 4 d was assumed to be ¼ of the total number of selected and dead broilers. Subsequently, feed intake until these days was estimated based on the performance objectives of each breed (Hubbard, 2017; Aviagen, 2018, 2019).

### Estimation of Greenhouse Gas Emissions of the Breeder, Hatchery, and Broiler Farm Stage

An attributional LCA was performed to estimate GHG emissions (CO_2_, CH_4_, and N_2_O) of the 3 different broiler systems for every stage in the broiler production chain. GHG emissions were expressed in kg CO_2_-equivalents based on their equivalent factor for 100 yr Global Warming Potential: 34 kg CO_2_-eq /kg biogenic CH_4_, 36.75 kg CO_2_-eq/ kg fossil CH_4_, and 298 kg CO_2_-eq / kg N_2_O (IPCC, 2013). For every stage in the broiler production chain, GHG emissions related to feed production, manure management on the farm, and energy use were estimated. Transport of eggs and chickens between breeder, hatchery, and broiler farm were excluded because of a relatively low impact on total GHG emissions and minor differences between systems.

#### Greenhouse Gas Emissions From Feed Production

Emissions of feed production were based on FeedPrint version 19.00 (Vellinga et al., 2013) and included emissions from crop cultivation, processing and drying of feed ingredients, transport to the feed mill and farm, and production of inputs (e.g., fertilizer, energy). Feed ingredients from the composed diets were matched with the available feed ingredients in FeedPrint, including the country of origin (Vellinga et al., 2013) (Appendix B, Table B.1). If there was no specific country for a feed ingredient, the default value for a combination of countries in FeedPrint, which is based on trade statistics, was selected. If the feed ingredient was not available in FeedPrint, a product with similar nutrient values was selected (Appendix B, Table B.1). Energy use at the feed mill and related GHG emissions was included based on FeedPrint. Emissions from land use change **(LUC)** from feed production were based on FeedPrint that estimated LUC with the PAS 2050:2011 method (BSI, 2011). This method is prescribed in the Product Environmental Footprint (**PEF**) guidelines (EC, 2018), which is recommended to use by industry in EU. Based on the feed ingredients in the diets, GHG emissions were estimated per kg feed of every diet in this study and emissions from LUC were expressed separately as recommended by the PEF guidelines. At broiler farms of the DRB and BLS production systems 20 kg of roughage (hay, straw, lucerne) per 1,000 birds is prescribed for these production systems (Saatkamp et al., 2019). In the model it was assumed that this roughage was artificially dried lucerne and the related GHG emissions were included.

#### Greenhouse Gas Emissions From Manure Storage and Energy Use on Farm

Emissions from manure storage on the farm were based on national inventory reports and IPCC (2006) and included direct N_2_O, indirect N_2_O (i.e., N_2_O derived from volatilization of NH_3_ and NO_x_), and CH_4_ emissions. For direct N_2_O, and indirect N_2_O emissions, nitrogen excretion was estimated based on nitrogen intake and nitrogen retained for growth and eggs production. Volatile solids were estimated to calculate CH_4_ emissions from manure. More details about calculation of emissions from manure can be found in Appendix C.

Emissions from energy use (natural gas and electricity) (kg CO_2_-eq/ MJ) were based on the production and use of an average energy mix for the Dutch situation from the ELCD database (JRC, 2018).

### Allocation of Emissions

At the laying period, economic allocation was performed to account for multiple output (eggs delivered to hatchery, non-hatching eggs, meat). In economic allocation, emissions are allocated to different products and co-products based on the economic value of these products. Prices of these products can be found in Appendix A, Table A.2. Also economic allocation was performed at the hatchery stage. It was assumed that unhatched eggs had no economic value, and therefore all emissions were allocated to hatched eggs. Number of eggs hatched at the hatchery was based on hatchability (Appendix A, Table A.4).

### Sensitivity Analyses

Input variables for the model in our study were based on production performances from industry and available literature. Our model did not account for farm variation in input variables and therefore we tested the sensitivity of the model to variation in input variables. Other LCA broiler studies (Leinonen et al., 2012; González-García et al., 2014; Wiedemann et al., 2017; Benavides et al., 2020) found that emissions from feed production and emissions from LUC from soybean production contributed most to the total GHG emissions at the broiler farm or slaughterhouse stage. Tallentire et al. (2017) also did an LCA broiler study and identified the inputs to which the environmental impact categories were most sensitive. For GHG emissions, these inputs were feed intake, age at slaughter, and slaughter weight. We varied the input of feed intake, slaughter weight, emissions from feed production, and emissions from LUC from soybean production. We did not vary age at slaughter, because this input has a minimum value in the DRB and BLS production system. No data were available about differences in variation between the production systems, and therefore we assumed that variation of input was similar in each production system.

In this analysis, all input parameters were adjusted separately and the impact on GHG emissions was compared with the results of reference situation (total GHG emissions at broiler farm gate in Table 3).

Variation in feed intake and slaughter weight can be found between broiler farms in the Netherlands (KWIN-V, 2018). Therefore, in the sensitivity analysis feed intake of broilers was increased and decreased by 5%, while having the same slaughter weight and diet compositions. Alternatively, slaughter weight of broilers was increased and decreased by 5%, while having the same feed intake and diet compositions. Emissions of feed production were changed by 25% based on Van Middelaar et al. (2013). Countries of origin of soybean products imported to the Netherlands differ over years (FAO, 2021). Therefore, country of soybean products (meal and oil) was changed from a country with high land use change (**HLUC**) emissions from soybean production (Brazil in this study) to a country with low land use change (**LLUC**) emissions from soybean production (USA in this study). It was assumed that the nutritional value of soybean products from countries with HLUC and LLUC emissions was similar.

## RESULTS

**Figure 2:**
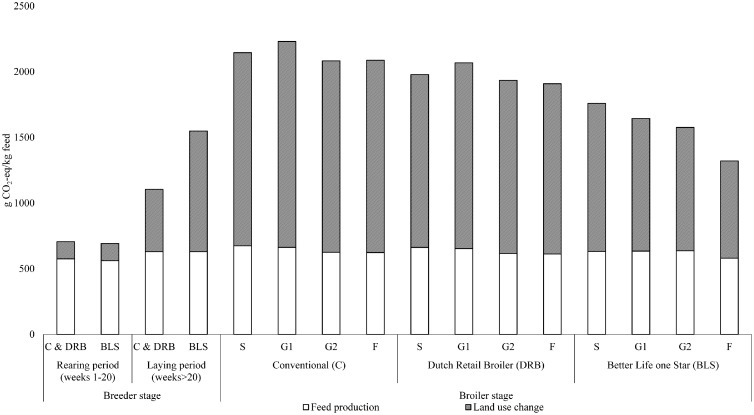
Greenhouse gas emissions (g CO_2_-eq /kg feed) of feed production and land use change missions from feed production for one diet for the rearing period, and one diet for the laying period, and starter (S), grower one (G1), grower two (G2), and finisher (F) diets for the broiler period for the conventional, Dutch Retail Broiler, and Better Life one Star broiler production systems.

The GHG emissions from feed production and LUC per kilogram feed were different at different stages of the broiler production chain (Figure 2). In the rearing period of the breeder stage, GHG emissions of feed production and LUC from feed production were similar for the production systems. In the laying period of the breeder stage, GHG emissions of feed production were similar for the production systems. However, emissions from LUC from feed production were higher in the BLS production system in the laying period due to a higher inclusion of soybean meal (**SBM**) to the diet. The country of origin of soybeans was Brazil in this study and these soybeans have high emissions from LUC (4,119 g CO_2_-eq/kg SBM) due to deforestation (Vellinga et al., 2013).

In the broiler farm stage, differences between the diets in GHG emissions were mainly due to LUC emissions from feed production. The highest difference in GHG emissions of feed production was between the starter diets of conventional (+ 40 g CO_2_-eq/kg feed) and BLS production system, whereas the highest difference in LUC emissions was between the finisher diet of the conventional (+ 720 g CO_2_-eq/kg feed) and BLS production system. This higher impact of LUC emissions was due to a higher percentage of SBM in the finisher diet of conventional compared to BLS.

Table 2 shows that the BLS production system has the lowest impact on GHG emissions per hen delivered to the laying period due to lowest feed intake and energy use per hen delivered (Appendix D, Table D.1). The highest impact on GHG emissions is from feed production for all production systems. The emissions per kg feed produced were similar for the production systems (Figure 2), and therefore, feed intake determines the most efficient production systems in the rearing period.

**Table 2:** Greenhouse gas emissions (kg CO_2_-eq) for the rearing period (0–20 wk) per hen delivered to the laying period, for the laying period (wk >20) per egg delivered to hatchery, and for the hatchery per egg hatched, for the three different Dutch broiler production systems.

	Conventional	Dutch retail broiler	Better life one Star
Rearing period			
Feed production	5.74	4.82	4.55
Land use change (LUC)^1^	1.30	1.09	1.06
Energy	2.17	2.08	2.06
Manure storage	0.38	0.32	0.34
Total	9.59	8.31	8.01
Total without LUC	8.29	7.22	6.95
Laying period			
Rearing period	0.06	0.05	0.04
Feed production	0.17	0.15	0.11
LUC	0.13	0.11	0.17
Energy	0.03	0.02	0.02
Manure storage	0.01	0.01	0.01
Total	0.40	0.34	0.35
Total without LUC^2^	0.26	0.22	0.17
Total after allocation	0.37	0.33	0.34
Hatchery			
Rearing and laying period	0.37	0.33	0.34
Unhatched eggs	0.07	0.06	0.06
Energy	0.06	0.06	0.06
Total	0.51	0.45	0.46
Total without LUC^3^	0.35	0.31	0.26

1 LUC emissions are related to feed production.

2 Without LUC from rearing period and from laying period.

3 Without LUC from rearing period and from laying period, and from losses by unhatched eggs.

The most efficient production system in the laying period is determined by the feed intake per egg delivered to hatchery and whether LUC emissions are included or excluded (Table 2). Feed intake per egg delivered to hatchery was lowest for the BLS production system (Appendix D, Table D.1). Emissions per kg feed produced were similar between the production systems, but LUC emissions per kg feed produced were different (Figure 2). When LUC emissions were excluded, the BLS production system had the lowest GHG emissions. When LUC emissions were included, the DRB production system had the lowest GHG emissions.

At the hatchery stage, hatchability was similar between the production systems (Appendix A, Table A.4), and therefore results showed the same pattern as in the laying period for the breeder stage (Table 2).

Table 3 shows GHG emissions of the 3 different production systems at the broiler farm gate per kg live weight. Main impact is from feed production and LUC from feed production (>50%) for all production systems. Feed intake and energy use per kg live weight produced was lowest for the conventional production system (appendix D, Table D.1), resulting in the lowest GHG emissions when LUC emissions from feed production were excluded. When LUC emissions from feed production were included, the BLS production system was the most efficient per kg live weight (Table 3).

**Table 3:** Greenhouse gas emissions (kg CO_2_-eq) expressed per kg live weight at the broiler farm gate for three different Dutch broiler production systems.

	Conventional	Dutch retail broiler	Better life one Star
Breeder^1^	0.15	0.14	0.14
Hatchery	0.05	0.05	0.05
Feed production	0.99	1.14	1.27
Land use change (LUC)^2^	2.30	2.43	1.80
Lucerne	0.00	0.01	0.01
Energy	0.12	0.16	0.21
Manure storage	0.04	0.05	0.06
Total	3.65	3.98	3.55
Total without LUC^3^	1.29	1.49	1.66

1 Breeder includes the laying and rearing period.

2 LUC emissions are related to feed production.

3 Without LUC from breeder, hatchery, and broiler farm stage.

### Sensitivity Analyses

**Figure 3:**
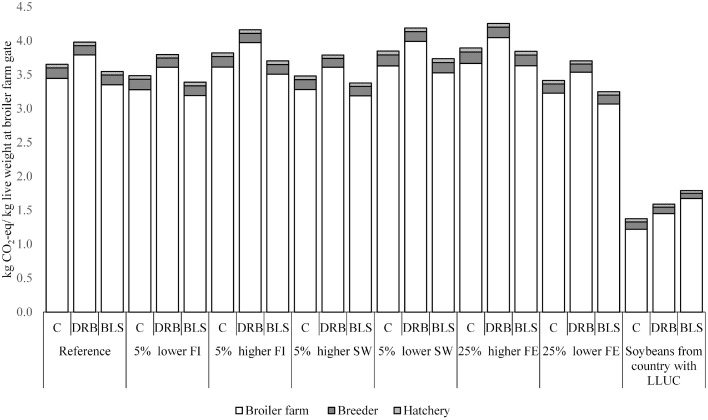
Results from sensitivity analyses showing the effect of a change in feed intake (FI), slaughter weight (SW), emissions from feed production (FE), and when soybeans in the diets were produced in country with low land use change (LLUC) emissions on greenhouse gas emissions in the breeder, hatchery, and broiler farm stage of the conventional (C), Dutch retail broiler (DRB), and Better Life one Star (BLS) broiler production system.

Results of the sensitivity analyses are shown in Figure 3. A 5% increased feed intake of broilers increased GHG emissions per kg live weight by approximately 4.5% for all broiler production systems, while a decreased feed intake had a similar but opposite effect. A 5% increased slaughter weight decreased GHG emissions for all production systems by 4.8%, while a decreased slaughter weight increased GHG emissions by 5.3%. Increased emissions related to feed production increased GHG emissions of conventional production system by 6.6%, DRB production system by 6.9%, and BLS production system by 8.4%. Decreased emissions related to feed production had a similar but opposite effect. Changing the country of origin of the soybeans from a HLUC to a LLUC country had a major impact on the results. The conventional production system (1.37 kg CO_2_-eq/ kg live weight) had the lowest emissions, whereas the BLS production system (1.79 kg CO_2_-eq/ kg live weight) had the highest emissions when the country of origin of soybeans changed from a HLUC to a LLUC country.

## DISCUSSION

Our study showed that the conventional, DRB, and BLS production systems were most efficient at different stages (Tables 2 and 3) of the broiler production chain. This shows the importance of including the whole chain in the assessment of GHG emissions of broiler production systems, and to implement specific mitigation options for different stages of the chain to reduce GHG emissions. In all stages, GHG emissions from feed production and emissions from LUC from feed production contributed most to the total.

Although there are no specific requirements to improve animal welfare in the breeder stage of the DRB and BLS production system, parent stock of slower growing DRB and BLS broilers have lower GHG emissions in both the rearing and laying period as compared to parent stock of conventional, in which fast-growing broiler chickens are used (Table 2). In the rearing period, the BLS system was most efficient, because feed intake (Appendix D, Table D.1), and mortality and selection were the lowest per hen delivered to the laying period (Appendix A, Table A.1.). GHG emissions per kg feed produced and emissions from LUC per kg feed produced were similar for all production systems and they contributed together for more than 70% to the total GHG emissions for all production systems. Therefore, emissions in the rearing period can be reduced by selecting feed ingredients with lower GHG emissions and reduce FCR in the rearing period. In the laying period, DRB (0.33 kg CO_2_-eq) and BLS (0.34 kg CO_2_-eq) were about 10% more efficient per egg delivered to the hatchery than the conventional production system (0.37 kg CO_2_-eq) (Table 2). Also in the laying period, the BLS production system had the lowest feed intake per egg delivered to hatchery (Appendix D, table D.1.). However, GHG emissions from LUC per kg feed produced in the laying period were higher in the BLS production system (Figure 2) and this had a high impact (50%) on total emissions in the laying period of the BLS production system. Therefore, in the laying period emissions can be reduced by selecting feed ingredients with lower GHG emissions, increase laying performance, and also by changing the country of origin for soybean products.

The contribution of the breeder and hatchery stages to the total GHG emissions at the broiler farm gate for all 3 production systems is about 5% and highest difference between production systems is about 0.01 kg CO_2_-eq/ kg live weight (Table 3). Although GHG emissions can be reduced in the breeder and hatchery stages, the highest reduction in the broiler production chain can be reached in the broiler farm stage, as this stage has the highest total impact on GHG emission. This was also found by Leinonen et al. (2012) who found a contribution of 8% and by Putman et al. (2017) who found a contribution of 4% from the breeder stage to total emissions at the broiler farm stage, for conventional broiler production systems.

At the broiler farm gate, BLS production system had the lowest GHG emissions. Emissions from feed production (>27%) and LUC from feed production contributed most (>50%) to total emissions (Table 3). Other LCA broiler studies (Boggia et al., 2010; Leinonen et al., 2012; González-García et al., 2014; Wiedemann et al., 2017) also found that emissions from feed production contributes most to the total GHG emissions. Emissions per kg feed produced were similar for the three production systems (Figure 2). However, FCR was the lowest in the conventional production system (Table 1), which resulted in the lowest emission from feed production per kg liveweight.

Emissions from LUC from feed production per kg liveweight were dependent on FCR and on emissions from LUC per kg feed produced. FCR was highest in BLS production system (Table 1). However, due to lower emissions from LUC per kg feed produced (Figure 2), total emissions were lowest for BLS production system. Soybean products are the main contributor to LUC emissions from feed production. BLS production system has a lower amount of soybean products in their diet due to lower nutritional recommendations compared to DRB and conventional production systems (Appendix A, Tables A.5–A.7).

Other LCA studies (Leinonen et al., 2012; Tallentire et al., 2018) found that other higher-welfare production systems, that is, free range and organic systems, or slower grower broilers had higher GHG emissions than the conventional production system or fast-growing chickens respectively. This was due to a higher feed consumption per bird in these systems or per slower grower broilers as compared to the conventional system or fast-growing broilers. Contrary to this, Boggia et al. (2010) found that the organic production system had lower GHG emissions than the conventional. Feed intake was higher in the organic system, but emissions for feed production were lower due to different feed composition of the diets.

Slaughter age, FCR, amount of soybean products in the diets, and emission from feed production, however, were different in these studies than our study. These differences make a comparison with results of our study difficult, but these studies (Boggia et al., 2010; Leinonen et al., 2012; Tallentire et al., 2018) also showed that FCR in combination with emissions from feed production determines which production system has the lowest impact. Moreover, our study showed that changing to indoor production systems with higher animal welfare not necessarily increases GHG emissions.

Input data in our study were based on performance objectives from industry and literature. Collecting primary data from broiler farmers, for example, about feed compositions and FCR may show variation in performance within and between production systems and thus variation in GHG emission. Our study, therefore, is an explorative study. Hence, some sensitivity analyses were performed to show the uncertainty of input. An increase of 5% of the slaughter weight or 5% reduced feed intake of broilers in the conventional production system, for example, resulted in lower GHG emissions of the conventional production system than the BLS production system without these changes. On broiler farms this variation in slaughter weight and feed intake exist (KWIN-V, 2018) which likely will result in a range in GHG emissions within production systems and overlap between production systems.

Emissions per feed ingredient are uncertain due to assumptions on input parameters such as yield, fertilization, and energy use to calculate these emissions (Van Middelaar et al., 2013). An increase or decrease of emissions related to feed production did not change conclusions about the productions system with the lowest impact. Due to the high contribution of GHG emissions from feed production to the total (>27%), a better estimation of these emissions can improve the estimation of GHG emissions of broiler production systems. LUC emissions from feed production had an important contribution to the total emissions (>53%) of the different broiler production systems. In our scenario with soy products in diets from a country with LLUC, the conventional system had lower GHG emissions than both ‘higher-welfare’ systems (DRB and BLS) (Figure 3). Soybeans are sourced from different countries to the Netherlands over years (FAO, 2021) and sourcing even may differ between broiler farmers. Therefore, which broiler system may have the lowest GHG emissions can differ over years. Thus, primary data from broiler farms, especially about feed intake, origin, and type of feed ingredients, and slaughter weight are required to show the variation between broiler farmers and production systems.

Currently, the Dutch broiler sector has no CO_2_-eq reduction targets in the Dutch climate agreement (Klimaatakkoord, 2019). This agreement, however, accounts only for GHG emissions that occur in the Netherlands, which are mainly emissions from manure and energy use for the broiler sector. The contribution of emissions from manure and energy use to the total at the broiler farm stage ranged from 4.3 to 7.3% which is low (Table 3). Main reduction of GHG emissions for the broiler sector can occur in emissions from feed production and LUC from feed production. However, most feed ingredients used in the Dutch broiler sector are produced in foreign countries. Therefore, reducing emissions from feed production and LUC from feed production will reduce GHG emissions worldwide and may contribute to reduction targets in other countries, but will have a minor impact on the Dutch reduction targets. Some Dutch retailers, however, do have CO_2_-eq reduction targets for the whole production chain of poultry production, for example, 15% reduction in 2030 compared to 2018 (AH, 2021). In addition, Dutch retailers will source their fresh broiler meat only from BLS production systems from 2023 onward (Dierenbescherming, 2021). Based on our calculations, changing from conventional to BLS production system would reduce GHG emissions by 2.9% and changing from DRB to BLS production system by 10.9%. Therefore, additional CO_2_-eq reductions are required to achieve this retailer target. Tallentire et al. (2017) showed that by optimizing diets on least GHG emissions, total GHG emissions were reduced by 37% in the UK and by 6.7% in the United States. Source of soybeans used in the diets was different between UK and United States and that resulted in large differences between emissions for production of SBM (3.05 kg in UK vs. 0.40 kg CO_2_-eq/kg SBM in United States), which largely impacted the reduction potential in that study. Our study showed that if soybeans were sourced from a country with LLUC or if LUC emissions were excluded, changing from conventional or DRB to BLS production system would increase GHG emissions by 12.6 to 30.3% (LLUC) or by 11.1% to 29.0% (excluding LUC) per kg liveweight (Table 3, Figure 3), which is unfavorable with respect to reduction targets.

A possible solution to meet reduction targets while maintaining broiler welfare, could be to use alternative feed ingredients in diets in the BLS system. Tallentire et al. (2018) showed that GHG emissions can be reduced by replacing soybean products completely with novel feed ingredients, such as microalgae, bacterial protein meal, or insect meal. In the analysis of Tallentire et al. (2018), however, emissions from LUC from soybean production were included and this may have affected conclusions about reduction potential of these novel ingredients. Both studies (Tallentire et al., 2017, 2018) showed that emissions from feed production can be reduced when GHG emissions constraints were included in formulation of diets of broilers. This can be extended by including additional constraints in formulation of diets to increase the use of alternative feed ingredients. These constraints can also contain environmental indicators from other environmental analyses than LCA such as an energy analysis. An energy analysis evaluates all of the natural resources depleted to create a product with a single unit of measure and can express how far a production system is from the full use of renewable resources (Castelleni et al., 2012). Castelleni et al. (2006) showed that organic crops saved around 60% energy compared to conventional crops by avoiding chemical fertilizers and pesticides. Moreover, also other indicators, such as land degradation, biodiversity losses, impact of pesticide use, that are currently not addressed in LCA should be considered (Van der Werf et al., 2020) and also other functions of agricultural systems, such as ecosystem services (e.g., landscape diversity), could be included (Van der Werf et al., 2020). Therefore, including additional constraints for selection of alternative feed ingredients in diets to increase environmental sustainability is complex and these constraints should be developed carefully.

Our study and results of Tallentire et al. (2017, 2018) showed the importance of including or excluding LUC emissions from soybean production for achieving the CO_2_-eq reduction targets. The PEF guidelines, which is recommend by European Commission to use by industry, states that LUC emissions from feed production should be reported separately (EC, 2018). However, retailers and other companies can decide by themselves whether these LUC emissions from feed production are included or excluded in CO_2_-eq reduction targets and including or excluding these emissions will give different mitigation options for broiler farmers. Moreover, this also questions whether CO_2_-eq reduction targets for every Dutch sector separately can reduce total GHG emissions in agricultural production. The definition of the system boundary, namely, determines whether total GHG emissions are reduced by changing to a different country of origin of soybeans used in the Dutch broiler sector (Frehner et al., 2020). When the system boundary is the Dutch broiler sector, this can be very effective to reduce GHG emissions. When the system boundary is broader, for example, the food or broiler sector worldwide, and assuming worldwide consumption of soybeans remains similar, the impact of changing to a different country of soybeans used in the Dutch broiler sector on GHG emissions is uncertain. In that case, emissions are displaced between sectors and that result in no reduction of total GHG emissions. Including these indirect consequences can be done by performing a consequential LCA while the PEF recommends an attributional LCA that excludes these indirect consequences. Therefore, by using an attributional LCA, caution should be taken with claiming reduction of GHG emissions by changing the country of origin from feed ingredients. The role of LUC emissions from feed production in claiming GHG emissions reductions should be considered in guidelines. This is important for retailers because our study showed that origin of and including or excluding LUC emissions from soybean production shows different conclusions for achieving the GHG emissions reduction targets set by retailers.

Although our study showed that changing toward ‘higher-welfare’ production systems can reduce GHG emissions, the impact on other environmental indicators was not analysed. Leinonen et al. (2012) and Tallentire et al. (2018), in their analysis, showed that other ‘higher-welfare’ broiler production systems increased agricultural land use, acidification potential, eutrophication potential, and primary energy use. This shows that other environmental indicators should also be considered, but also that having reduction targets for several environmental indicators simultaneously can become very difficult.

Having targets for GHG emissions is a good starting point for the broiler sector, and this should be expanded with other environmental indicators in the future.

## Conclusions

This study showed that changing towards 'higher-welfare’ broiler production systems can reduce GHG emissions. At the breeder stage, the DRB and BLS broiler production system had the lowest GHG emissions. At the broiler farm stage, BLS production system (3.55 kg CO_2_-eq/kg live weight) had lower GHG emissions compared to conventional (3.65 kg CO_2_-eq/kg live weight) and DRB (3.98 kg CO_2_-eq/kg LW) production system. Sensitivity analyses showed that variation in slaughter weight and feed intake could result in overlap of GHG emissions between production systems. Moreover, when soybean products were sourced from a country with low LUC emissions, the conventional production system (1.37 kg CO_2_-eq/ kg live weight) had the lowest GHG emissions and the BLS production system (1.79 kg CO_2_-eq/kg live weight) the highest. These results are not only relevant for the current case study on Dutch broiler production systems, but also in light of the development toward an increasing number of higher-welfare broiler production systems in Europe (ECC, 2018). Primary data from broiler farms, especially about feed intake, origin and type of feed ingredients, and slaughter weight are required to show the variation among farmers and production systems and to estimate whether GHG emissions reduction targets can be achieved. In addition, this study showed that origin of and including or excluding LUC emissions from soybean production shows different conclusions for achieving the GHG emissions reduction targets set by retailers.

## Appendix Supplementary materials

Image, application 1

## Footnotes

Supplementary material associated with this article can be found, in the online version, at doi:10.1016/j.psj.2022.102151.
