# Supplementary material for: The impact of changing toward higher welfare broiler production systems on greenhouse gas emissions: a Dutch case study using life cycle assessment
Source: Poult Sci. 2022 Aug 27;101(12):102151. doi: 10.1016/j.psj.2022.102151 (PMC9593752; doi:10.1016/j.psj.2022.102151)
Supplement: Supplementary file 1 [file mmc1.docx]

**Appendix A**

**Table A.1.** Input of the rearing period of the conventional (Ross 308), Dutch Retail Broiler (Ranger Classic), and Better Life one Star (Hubbard JA 257) broiler production systems to estimate greenhouse gas emissions of these broiler production systems

| Broiler breed | Ross 308^c^ | Ranger Classic ^c^ | Hubbard JA257 ^c^ |
| --- | --- | --- | --- |
| Chicken start (chicken/m^2^)^a^ | 9.5 | 9.5 | 9.5 |
| Mortality and selection (%)^b^ | 9.0 | 5.0 | 4.0 |
| Chicken to laying period (chicken/m^2^) | 8.6 | 9.0 | 9.1 |
| Feed intake (females only) ^b^ |  |  |  |
| Starter 1 (0-20 days, kg) | 0.57 | 0.54 | 0.46 |
| Starter 2 (21-34 days, kg) | 0.55 | 0.54 | 0.54 |
| Grower (35-104 days, kg) | 4.32 | 3.74 | 3.75 |
| Pre Breeder (105 – 139 days, kg) | 3.28 | 2.56 | 2.40 |
| Weight hen at 20 weeks (kg) ^b^ | 2.34 | 1.97 | 1.59 |
| Feed intake (males only) ^b^ |  |  |  |
| Starter 1 (0-20 days, kg) | 0.61 | 0.61 | 0.62 |
| Starter 2 (21-34 days, kg) | 0.70 | 0.70 | 0.71 |
| Grower (35-104 days, kg) | 5.40 | 5.40 | 5.45 |
| Pre Breeder (105 – 139 days, kg) | 3.22 | 3.22 | 3.25 |
| Male weight at 20 weeks (kg) ^b^ | 3.04 | 3.04 | 3.08 |
| Electricity (€/100 chickens housed) ^a^ | 10 | 10 | 10 |
| Gas (€/100 chickens housed) ^a^ | 31 | 31 | 31 |

^a^ KWIN-V, 2018

^b^ Aviagen, 2016, 2018; Hubbard 2019

^c^ Parent males for the broiler breeds were the following: Ross male (Ross 308), Ross male (Ranger Classic), Hubbard M22 (Hubbard JA257)

Parent females for the broiler breeds were the following: Ross 308 female (Ross 308), Ranger female (Ranger Classic), Hubbard JA57 (Hubbard JA257)

**Table A.2.** Input of the laying period of the conventional (Ross 308), Dutch Retail Broiler (Ranger Classic) and Better Life one Star (Hubbard JA 257) broiler production systems to estimate greenhouse gas emissions of these broiler production systems

| Broiler production systems | Conventional | Better Life one Star | | |
| --- | --- | --- | --- | --- |
| Broiler breed | Ross 308^c^ | Ranger Classic ^c^ | | Hubbard JA257 ^c^ |
| Number of hen at start (hen/m^2^)^a,b^ | 7.0 | 7.0 | 7.6 | |
| Mortality and selection(%)^b^ | 8.0 | 8.0 | 6.0 | |
| Mating ratio (%)^b^ | 8.0 | 8.0 | 8.0 | |
| Depletion age (weeks) ^b^ | 60 | 60 | 65 | |
| Total eggs per hen housed (#)^b^ | 177 | 184 | 233 | |
| Non hatching eggs per hen housed (#)^b^ | 8 | 11 | 12 | |
| Total hatching eggs per hen housed (#)^b^ | 169 | 173 | 221 | |
| Feed intake (females only)^b^ |  |  |  | |
| Pre breeder 20-23 weeks (kg) | 3.4 | 2.6 | 2.2 | |
| Breeder one 24-60 weeks (kg) | 41.9 | 37.1 |  | |
| Breeder one 24-65 weeks (kg) |  |  | 35.6 | |
| Weight hen at depletion age (kg) | 4.08 | 3.27 | 2.24 | |
| Feed intake (males only) ^b^ |  |  |  | |
| Pre breeder 20-23 weeks (kg) | 2.9 | 2.9 | 2.9 | |
| Breeder one 24-60 weeks (kg) | 34.8 | 34.8 |  | |
| Breeder one 24-65 weeks (kg) |  |  | 40.2 | |
| Weight male at depletion age (kg)^b^ | 5.00 | 5.00 | 5.05 | |
| Electricity (€/100 chickens) ^a^ | 56 | 56 | 62 | |
| Gas (€/100 chickens) ^a^ | 18 | 18 | 20 | |
| Price meat (€/kg chicken) | 0.4 | 0.4 | 0.4 | |
| Price per hatching egg (€/egg) | 0.185 | 0.185 | 0.185 | |
| Price non hatching egg (€/egg) | 0.02 | 0.02 | 0.02 | |

^a^ KWIN-V, 2018

^b^ Aviagen, 2016, 2018; Hubbard 2019

^c^ Parent males for the broiler breeds were the following: Ross male (Ross 308), Ross male (Ranger Classic), Hubbard M22 (Hubbard JA257)

Parent females for the broiler breeds were the following: Ross 308 female (Ross 308), Ranger female (Ranger Classic), Hubbard JA57 (Hubbard JA257)

**Table A.3.** Diet composition (%/kg) and nutritional constraints of the rearing period (weeks 1-20) and laying period (weeks> 20) of the conventional (Ross 308), Dutch Retail Broiler (Ranger Classic) and Better Life one Star (Hubbard JA 257) broiler production systems

| Broiler breed^a^ | Ross308, Ranger Classic | Ross308, Ranger Classic | Hubbard JA257 | Hubbard JA257 |
| --- | --- | --- | --- | --- |
|  | Weeks 1-20 | Weeks >20 | Weeks 1-20 | Weeks >20 |
| Corn | 44.40 | 41.88 | 29.85 | 32.34 |
| Wheat | 9.95 | 24.87 | 24.03 | 30.36 |
| Sunflower seed meal | 0.00 | 14.86 | 4.94 | 10.93 |
| Limestones |  | 6.53 |  | 7.12 |
| Soybean meal 48.5-50% CP | 0.00 | 5.33 | 0.00 | 14.48 |
| Rapeseed extr. pou | 2.75 | 2.49 | 6.97 | 0.00 |
| Soy bean oil crude | 0.50 | 1.49 | 0.50 | 2.16 |
| Bonephosphate (DCP) | 1.11 | 0.70 | 0.68 | 1.03 |
| Prophorce sa exclusive | 0.60 | 0.60 | 0.60 | 0.60 |
| px SLF-1ZC 0.25% | 0.30 | 0.30 | 0.30 | 0.30 |
| Salt stone | 0.24 | 0.27 | 0.15 | 0.25 |
| L-lysine sulphate 55% | 0.29 | 0.25 | 0.32 | 0.13 |
| L-methionine 99% | 0.12 | 0.13 | 0.05 | 0.11 |
| Xyl/Fyt-mix 0.10% | 0.10 | 0.10 | 0.10 | 0.10 |
| Sunfl.extracted 29 | 16.92 | 0.07 | 8.65 | 0.00 |
| L-threonine 99% powder | 0.08 | 0.06 | 0.01 | 0.00 |
| Na bicarbonate | 0.05 | 0.05 | 0.05 | 0.05 |
| Cholin chlorid 75% liquid | 0.04 | 0.04 | 0.04 | 0.04 |
| Barley | 2.99 | 0.00 | 2.99 | 0.00 |
| Nutricell pellet | 4.00 | 0.00 | 4.00 | 0.00 |
| Lime fine | 0.82 | 0.00 | 0.93 | 0.00 |
| Wheat grits | 14.77 | 0.00 | 14.87 | 0.00 |
| Nutrients of diet |  |  |  |  |
| ME Poultry (Kcal) | 2600 | 2800 | 2600 | 2800 |
| Gross energy (MJ) | 16.04 | 15.50 | 16.12 | 15.55 |
| Crude Protein (%) | 13.11 | 14.94 | 14.47 | 16.80 |
| Moisture (%) | 11.31 | 10.87 | 11.32 | 10.79 |
| Undigestible CP(%) | 2.43 | 2.39 | 2.80 | 2.52 |
| Digestible Lys (%) | 0.52 | 0.6 | 0.59 | 0.69 |
| Calcium (%) | 0.90 | 3.00 | 0.86 | 3.30 |
| Available Phosphorus (%) | 0.42 | 0.35 | 0.36 | 0.41 |

^a^ Parent males for the broiler breeds were the following: Ross male (Ross 308), Ross male (Ranger Classic), Hubbard M22 (Hubbard JA257)

Parent females for the broiler breeds were the following: Ross 308 female (Ross 308), Ranger female (Ranger Classic), Hubbard JA57 (Hubbard JA257)

**Table A.4.** Input to estimate greenhouse gas emissions of the hatchery of the conventional (Ross 308), Dutch Retail Broiler (Ranger Classic) and Better Life one Star (Hubbard JA 257) broiler production systems

| Broiler breed | Ross 308 | Ranger Classic | Hubbard JA257 |
| --- | --- | --- | --- |
| Hatchability (%)^a^ | 83.7 | 84.3 | 84.4 |
| Chicken hatched (#) | 142 | 146 | 186 |
| Energy costs (€/1000 eggs housed) ^b^ | 12.5 | 12.5 | 12.5 |

^a^ Aviagen, 2016, 2018; Hubbard 2018

^b^ KWIN-V, 2018

**Table A.5.** Diet composition (%/kg) of the conventional production system (Ross 308) for starter, grower 1, grower 2, and finisher diets.

| Ingredient | Starter  (day 0-10) | Grower 1  (day 11-20) | Grower 2  (day 21-30) | Finisher  (day 31-38) |
| --- | --- | --- | --- | --- |
| Wheat | 22.96 | 24.88 | 34.83 | 44.78 |
| Corn | 34.83 | 33.25 | 19.14 | 14.93 |
| Breadmeal | 0.00 | 1.99 | 7.96 | 7.96 |
| Oats | 0.00 | 0.00 | 0.00 | 0.00 |
| Soybean meal 48.5-50% CP | 29.30 | 27.74 | 22.65 | 22.16 |
| Potato protein | 1.50 | 0.00 | 0.00 | 0.00 |
| Rapeseedmeal 00 | 0.00 | 0.00 | 2.99 | 1.99 |
| Fieldbeans (white) | 4.98 | 4.98 | 4.98 | 0.66 |
| Sunflower meal 35-38% CP | 0.00 | 0.00 | 0.00 | 0.00 |
| Nutricell pellet | 1.00 | 1.00 | 1.00 | 1.00 |
| Soy bean oil crude | 1.70 | 2.98 | 3.80 | 4.04 |
| Lime fine | 0.94 | 0.84 | 0.79 | 0.75 |
| Bonephosphate porcine (DCP) | 1.11 | 0.76 | 0.53 | 0.38 |
| Na bicarbonate | 0.15 | 0.15 | 0.15 | 0.15 |
| Salt stone | 0.27 | 0.24 | 0.14 | 0.14 |
| L-lysine sulphate 55% | 0.37 | 0.35 | 0.33 | 0.35 |
| L- methionine 99% | 0.34 | 0.30 | 0.26 | 0.23 |
| L-threnine 99% powder | 0.13 | 0.12 | 0.10 | 0.10 |
| Valine 40% PX | 0.05 | 0.05 | 0.01 | 0.01 |
| Cholin chlorid 75% liquid | 0.04 | 0.04 | 0.04 | 0.04 |
| Broiler premix 0.3% | 0.30 | 0.30 | 0.30 | 0.30 |
| Wheat enzyme | 0.03 | 0.03 | 0.03 | 0.03 |
| Fytase | 0.02 | 0.02 | 0.02 | 0.02 |
| Nutrients of diet |  |  |  |  |
| ME Poultry (Kcal) | 3000 | 3100 | 3150 | 3200 |
| Gross energy (MJ) | 16.67 | 16.96 | 17.22 | 17.24 |
| Crude Protein (%) | 22.3 | 20.67 | 20.05 | 19.09 |
| Moisture (%) | 11.58 | 11.47 | 11.3 | 11.31 |
| Undigestible CP (%) | 3.04 | 2.84 | 2.92 | 2.77 |
| Digestible Lysine (%) | 1.25 | 1.12 | 1.04 | 0.98 |
| Calcium (%) | 0.96 | 0.84 | 0.78 | 0.72 |
| Available Phosphorus (%) | 0.48 | 0.42 | 0.39 | 0.36 |

**Table A.6.** Diet composition (%/kg) of the Dutch Retail Broiler production system (Ranger Classic) for starter, grower 1, grower 2, and finisher diets

| Ingredient | Starter  (day 0-10) | Grower 1  (day 11-20) | Grower 2  (day 21-30) | Finisher  (day 31-49) |
| --- | --- | --- | --- | --- |
| Wheat | 27.36 | 27.00 | 34.83 | 44.78 |
| Corn | 33.44 | 34.04 | 22.60 | 16.77 |
| Breadmeal | 0.01 | 1.99 | 7.96 | 7.96 |
| Oats | 0.00 | 0.00 | 0.00 | 0.00 |
| Soybean meal 48.5-50% CP | 26.68 | 25.24 | 21.32 | 19.65 |
| Potato protein | 1.50 | 0.00 | 0.00 | 0.00 |
| Rapeseedmeal 00 | 0.00 | 0.00 | 1.49 | 1.00 |
| Fieldbeans (white) | 4.98 | 4.98 | 4.98 | 2.82 |
| Sunflower meal 35-38% CP | 0.00 | 0.00 | 0.00 | 0.00 |
| Nutricell pellet | 1.00 | 1.00 | 1.00 | 1.00 |
| Soy bean oil crude | 1.35 | 2.57 | 3.13 | 3.55 |
| Lime fine | 0.95 | 0.85 | 0.81 | 0.77 |
| Bonephosphate porcine (DCP) | 1.12 | 0.78 | 0.56 | 0.39 |
| Na bicarbonate | 0.15 | 0.15 | 0.15 | 0.15 |
| Salt stone | 0.27 | 0.24 | 0.14 | 0.14 |
| L-lysine sulphate 55% | 0.37 | 0.35 | 0.33 | 0.34 |
| L- methionine 99% | 0.31 | 0.28 | 0.24 | 0.21 |
| L-threnine 99% powder | 0.12 | 0.11 | 0.09 | 0.09 |
| Valine 40% PX | 0.03 | 0.05 | 0.00 | 0.01 |
| Cholin chlorid 75% liquid | 0.04 | 0.04 | 0.04 | 0.04 |
| Broiler premix 0.3% | 0.30 | 0.30 | 0.30 | 0.30 |
| Wheat enzyme | 0.03 | 0.03 | 0.03 | 0.03 |
| Fytase | 0.02 | 0.02 | 0.02 | 0.02 |
| Nutrients of diet | |  |  |  |
| ME Poultry (Kcal) | 3000 | 3100 | 3150 | 3200 |
| Gross energy (MJ) | 16.54 | 16.82 | 17.02 | 17.08 |
| Crude Protein (%) | 21.40 | 19.76 | 19.13 | 18.24 |
| Moisture (%) | 11.67 | 11.56 | 11.42 | 11.41 |
| Undigestible CP (%) | 2.93 | 2.72 | 2.73 | 2.60 |
| Digestible Lysine (%) | 1.19 | 1.07 | 0.99 | 0.93 |
| Calcium (%) | 0.96 | 0.84 | 0.78 | 0.72 |
| Available Phosphorus (%) | 0.48 | 0.42 | 0.39 | 0.36 |

**Table A.7.** Diet composition (%/kg) of the Better Life one Star production system (Hubbard JA257) for starter, grower 1, grower 2, and finisher diets

| Ingredient | Starter  (day 0-14) | Grower 1  (day 15-25) | Grower 2  (day 26-35) | Finisher  (day 36-56) |
| --- | --- | --- | --- | --- |
| Wheat | 21.01 | 27.89 | 34.83 | 44.78 |
| Corn | 34.83 | 34.83 | 32.43 | 18.02 |
| Breadmeal | 0.00 | 1.99 | 0.21 | 7.96 |
| Oats | 4.81 | 1.06 | 0.00 | 0.00 |
| Soybean meal 48.5-50% CP | 23.33 | 18.90 | 15.16 | 10.14 |
| Potato protein | 0.00 | 0.00 | 0.00 | 0.00 |
| Rapeseedmeal 00 | 0.00 | 0.00 | 2.99 | 5.97 |
| Fieldbeans (white) | 4.98 | 4.98 | 3.67 | 4.98 |
| Sunflower meal 35-38% CP | 4.98 | 4.98 | 4.98 | 3.05 |
| Nutricell pellet | 2.00 | 1.00 | 1.00 | 1.00 |
| Soy bean oil crude | 0.94 | 1.44 | 2.02 | 2.13 |
| Lime fine | 0.76 | 0.68 | 0.60 | 0.48 |
| Bonephosphate porcine (DCP) | 0.94 | 0.65 | 0.53 | 0.24 |
| Na bicarbonate | 0.15 | 0.15 | 0.15 | 0.15 |
| Salt stone | 0.27 | 0.23 | 0.26 | 0.11 |
| L-lysine sulphate 55% | 0.33 | 0.43 | 0.45 | 0.39 |
| L- methionine 99% | 0.25 | 0.26 | 0.22 | 0.17 |
| L-threnine 99% powder | 0.06 | 0.10 | 0.09 | 0.08 |
| Valine 40% PX | 0.00 | 0.05 | 0.05 | 0.00 |
| Cholin chlorid 75% liquid | 0.04 | 0.04 | 0.04 | 0.04 |
| Broiler premix 0.3% | 0.30 | 0.30 | 0.30 | 0.30 |
| Wheat enzyme | 0.03 | 0.03 | 0.03 | 0.03 |
| Fytase | 0.02 | 0.02 | 0.02 | 0.02 |
| Nutrients of diet | |  |  |  |
| ME Poultry (Kcal) | 2900 | 3025 | 3058 | 3100 |
| Gross energy (MJ) | 16.46 | 16.57 | 16.67 | 16.80 |
| Crude Protein (%) | 20.25 | 18.83 | 18.00 | 17.24 |
| Moisture (%) | 11.59 | 11.62 | 11.63 | 11.58 |
| Undigestible CP (%) | 2.86 | 2.66 | 2.68 | 2.71 |
| Digestible Lysine (%) | 1.05 | 1.00 | 0.95 | 0.85 |
| Calcium (%) | 0.85 | 0.75 | 0.70 | 0.60 |
| Available Phosphorus (%) | 0.45 | 0.40 | 0.38 | 0.34 |

**References**

Aviagen. 2016. ROSS 308 European Parent stock: Performance Objectives, 2016. Aviagen Group, Huntsville, AL 35806 USA.

Aviagen. 2018. Ranger Classic Parent stock Performance Objectives, 2018. Aviagen Group, Huntsville, AL 35806 USA.

Hubbard. 2019. Performance objectives parent stock JA57, V-05-2019. Hubbard, PIKEVILLE, TN 37367, USA.

KWIN-V. 2018. Quantitative Livestock Farming Information 2017-2018 (Kwantitatieve Informatie Veehouderij 2017-2018). Livestock Research, Wageningen UR, the Netherlands.

**Appendix B**

**Table B.1.** Greenhouse gas emissions of feed production, land use change and total of different feed ingredients per kilogram product (Vellinga et al., 2013)

|  |  | Land use change | Feed production | Total |
| --- | --- | --- | --- | --- |
| Ingredient | Ingredient FeedPrint | (g CO_2_ eq/kg) | (g CO_2_ eq/kg) | (g CO_2_ eq/kg) |
| Corn | Maize Ukraine | 112 | 535 | 647 |
| Wheat | Wheat France | 30 | 416 | 445 |
| Sunflower seed meal | Sunflower seed meal, CF 160-200 | 19 | 467 | 486 |
| Limestones | Chalk grit | 0 | 519 | 519 |
| Soybean meal 48.5-50% CP | Soybean meal, CF 0-45, CP >480 Brasil Crush NL | 4119 | 599 | 4718 |
| Rapeseed extr. pou | Rape seed meal solvent extracted, CP 0-380 | 300 | 502 | 802 |
| Soy bean oil crude | Fat/oil, Soya oil Brasil | 12734 | 1688 | 14423 |
| Bonephosphate (DCP) | Monocalciumphosphate | 0 | 575 | 575 |
| Prophorce sa exclusive | Lactic acid (100% liquid) | 32 | 3168 | 3200 |
| px SLF-1ZC 0.25% | Mineral mix | 8 | 1093 | 1101 |
| Salt stone | Salt | 0 | 180 | 180 |
| L-lysine sulphate 55% | L-Lysin HCL | 56 | 6437 | 6493 |
| L-methionine 99% | DL-Methionin | 0 | 3050 | 3050 |
| Xyl/Fyt-mix 0.10% | Fytase 1 (max 0.20%) | 112 | 12865 | 12977 |
| Sunfl.extracted 29 | Sunflower seed meal, CF >240 | 16 | 403 | 419 |
| L-threonine 99% powder | L-Threonin | 56 | 6437 | 6493 |
| Na bicarbonate | Sodiumbicarbonate | 1 | 490 | 491 |
| Cholin chlorid 75% liquid | Vitamin mix | 52 | 6351 | 6403 |
| Barley | Barley | 42 | 396 | 438 |
| Nutricell pellet | Oats husk meal | 1 | 232 | 233 |
| Lime fine | Chalk (finely milled) | 0 | 1225 | 1225 |
| Wheat grits | Wheat middlings | 11 | 270 | 281 |
| Breadmeal | Bread meal | 0 | 124 | 124 |
| Oats | Oats grain | 2 | 497 | 498 |
| Potato protein | Potato protein, ash 0-10 | 0 | 1316 | 1316 |
| Rapeseedmeal 00 | Rape seed meal solvent extracted, CP 0-380 | 300 | 502 | 802 |
| Fieldbeans (white) | Horse beans white | 0 | 402 | 402 |
| Sunflower meal 35-38%CP | Sunflower seed meal, CF 160-200 | 19 | 467 | 486 |
| Nutricell Pallet | Oats husk meal | 1 | 232 | 233 |
| Valine 40% PX | L-Valin | 52 | 6351 | 6403 |
| Cholin chlorid 75% liquid | Vitamin mix | 52 | 6351 | 6403 |
| Broiler premix 0.3% | Mineral mix | 8 | 1093 | 1101 |
| Wheat enzyme | Mineral mix | 8 | 1093 | 1101 |
| Fytase | Fytase 1 (max 0.20%) | 112 | 12865 | 12977 |
| Lucerne | Lucerne (alfalfa) artificially dried | 0 | 1455 | 1455 |
| Scenario |  |  |  |  |
| Soybean meal 48.5-50% CP | Soybean meal, CF 0-45, CP >480 USA Crush NL | 14 | 613 | 627 |
| Soy bean oil crude | Fat/oil, Soya oil USA | 43 | 1731 | 1774 |

**References**

Vellinga T.V., H. Blonk, M. Marinussen, W.J. Van Zeist, and I.J.M. De Boer. 2013. Methodology used in feedprint: a tool quantifying greenhouse gas emissions of feed production and utilization. Version 19.00. Wageningen UR Livestock research, Lelystad, the Netherlands.

**Appendix C**

**Calculation of direct and indirect N_2_O emissions**

To calculate direct and indirect N_2_O emissions, first N and TAN excreted were estimated. Second, based on the N and TAN excretion, direct and indirect N_2_O missions were calculated

N excreted= N intake from feed –N retained

Where:

N intake is estimated based on kg feed intake (Table 1, Table A.1, Table A.2) and N content of the diets

N content of diets was crude protein x 0.16 (Table A.3, Table A.5 – A.7)

N retained is based on kg N retained in meat and eggs (Table C.1)

**Table C.1.** Nitrogen content of parent stock, broilers and eggs (CBS, 2018)

|  | N (g/kg live weight) |
| --- | --- |
| Day-old chicken | 25.8 |
| Parent female 20 weeks | 33.4 |
| Parent female end of life | 28.4 |
| Parent male 20 weeks | 34.5 |
| Parent male end of life | 35.4 |
| Broiler | 28.3 |
| Eggs | 19.3 |

TAN was estimated by first multiplying N intake with the N digestibility (based on crude protein (%/kg) and undigestible crude protein (%/kg) in the diets, Table A.3, Table A.5 – A.7) and second subtracting the N retained.

**Direct N_2_O Emissions**

Direct N_2_O Emissions (IPCC 2006) were estimated by:

N excreted x EFN_2_O x 44/28

Where:

EFN_2_O is emission factors in kg N_2_O-N/kg N (0.001)

44/28 conversion from N_2_O-N to N_2_O

**Indirect N_2_O emissions**

Indirect N_2_O emissions (IPCC 2006) due to volatilisation were estimated by:

(NH_3_ x 14/17+NO_X_-N) x EFNH_3_NO_x_ x 44/28

Where:

EFNH_3_NO_x_ is emission factor for N_2_O emissions from atmospheric deposition of nitrogen on soils and water surfaces, kg N_2_O-N (kg NH_3_-N + NO_x_-N volatilized); default value is 0.01 kg N_2_O-N (kg NH_3_-N + NO_x_-N volatilized)

14/17 is conversion of NH_3_ to NH_3_-N

**NH_3_ emissions**

Emission factors of NH_3_ were based on national inventory reports (Van Bruggen et al., 2018). Emission factors were based on the following housing systems: other low emissions housing for the rearing period, floor housing with manure aeration from above for the laying period, and mixed air ventilation, heaters and fans for broiler farms.

NH_3_ was estimated by:

TAN x EFNH_3_ x 17/14

Where

EFNH_3_ is emission factor NH_3_-N for parents and broilers (Table C.2)

17/14 conversion of NH_3_-N to NH_3_

**Table C.2.** Emissions factors of NH_3_-N of the rearing period, laying period, and broiler farms (Van Bruggen et al., 2018)

|  | Rearing period | Laying period | Broiler farm |
| --- | --- | --- | --- |
| EF N-NH_3_ (%/TAN) | 20.3 | 21.2 | 5.5 |

**NO_x_ emissions**

NO_x_-N was estimated by:

N excreted x EFNO_x_

Where:

EFNO_x_ is emission factors in kg NO_x_-N/kg N (0.001) (IPCC, 2006)

**Calculation of CH_4_ emissions**

To calculate CH_4_ emissions, first volatile solids were calculated. Second CH_4_ emissions were calculated.

Volatile solids was estimated by (FAO, 2017):

DM x (1- ME_feed_/GE_feed_) x (1-0.3)

Where:

DM is dry matter intake (kg) estimated based on moisture content (Table A.3, Table A5 - A10)

ME_feed_ is metabolic energy of feed (MJ) (Table A.3, Table A.5 – A.7)

GE_feed_ is gross energy of feed (MJ) (Table A.3, Table A.5 – A.7)

0.3 is ash content of manure

Methane emissions were estimated by the following formulas (IPCC 2006):

VS x Bo x MCF x methane density

Where:

VS is volatile solids (kg)

Bo is maximum methane production potential (0.34 m^3^ CH_4_/kg VS) for the manure

MCF is methane conversion factor for livestock category and manure management system (1.5%)

Methane density is 0.67 kg/m^3^ CH_4_

**References**

CBS. 2018. Dierlijke mest en mineralen 2017. Centraal Bureau voor de Statistiek, Den Haag/Heerlen/Bonaire, 2018.

FAO. 2017. GLOBAL LIVESTOCK ENVIRONMENTAL ASSESSMENT MODEL. Model Description Version 2.0 Food and Agriculture Organization of the United Nations, Rome, Italy.

IPCC. 2006. Intergovernmental Panel on Climate Change. Guidelines for national greenhouse gas inventories, in: H.S. Eggleston, L. Buendia, K. Miwa, T. Ngara, K. Tanabe (Eds.), Agriculture, Forestry and Other Land Use, vol. 4, IGES, Japan (2006)

Van Bruggen, C., A. Bannink, C.M. Groenestein, J.F.M. Huijsmans, L.A. Lagerwerf, H.H. Luesink, S.M. van der Sluis, G.L. Velthof, and J. Vonk. 2018. *Emissies naar lucht uit de landbouw in 2016. Berekeningen met het model NEMA.* WOT Natuur & Milieu, WOt-technical report 119, Wageningen, the Netherlands.

**Appendix D**

**Table D.1.** Production parameters of the conventional (Ross 308), Dutch Retail Broiler (Ranger Classic) and Better Life one Star (Hubbard JA 257) broiler production systems in the rearing, laying, and broiler farm stage expressed in different functional units

| Production parameters | Ross 308 | Ranger Classic | Hubbard JA257 |
| --- | --- | --- | --- |
| Rearing period (per hen delivered to laying period) |  |  |  |
| Feed (kg) | 9.985 | 8.390 | 8.118 |
| Electricity (kWh) | 0.913 | 0.874 | 0.865 |
| Gas (m^3^) | 0.669 | 0.641 | 0.634 |
| Laying period (per egg delivered to hatchery) |  |  |  |
| Feed (kg) | 0.274 | 0.237 | 0.181 |
| Electricity (kWh) | 0.027 | 0.027 | 0.023 |
| Gas (m^3^) | 0.002 | 0.002 | 0.002 |
| Broiler farm (per kilogram live weight at farm gate) |  |  |  |
| Feed (kg) | 1.553 | 1.842 | 2.105 |
| Electricity (kWh) | 0.070 | 0.093 | 0.117 |
| Gas (m^3^) | 0.029 | 0.040 | 0.056 |
